# Fugitives on the run: circulating tumor cells (CTCs) in metastatic diseases

**DOI:** 10.1007/s10555-019-09795-4

**Published:** 2019-05-03

**Authors:** Tania Mamdouhi, Julianne D. Twomey, K. Melodi McSweeney, Baolin Zhang

**Affiliations:** Office of Biotechnology Products, Center for Drug Evaluation and Research, Food and Drug Administration, Silver Spring, MD 20993 USA

**Keywords:** Circulating tumor cells, Metastasis, Liquid biopsy, Biomarker

## Abstract

The presence of circulating tumor cells (CTCs) in the bloodstream signals the existence of a tumor and denotes risk of metastatic spread. CTCs can be isolated and analyzed to monitor cancer progression and therapeutic response. However, CTC isolation devices have shown considerable variation in detection rates, limiting their use as a routine diagnostic and monitoring tool. In this review, we discuss recent advances in CTC detection methodologies and associated clinical studies. We provide perspective on the future direction of CTC isolation and molecular characterization towards developing reliable biomarkers that monitor disease progression or therapeutic response.

## Introduction

The metastatic spread of cancer cells from a primary tumor to distant sites is the leading cause of mortality in cancer patients [1]. The underlying molecular mechanisms of metastasis are not well understood and remain a fundamental topic in the field of cancer research. The isolation and characterization of tumor cells that have broken off from the original tumor site and circulate within the peripheral blood stream can provide insight into the process of metastatic spread and can relay a real-time monitoring of disease progression and therapeutic response. This characterization of circulating tumor cells (CTCs) holds promise for guiding personalized therapies and developing novel anticancer drugs specifically targeting the metastatic process. CTCs exist in extremely low quantities in the blood stream, making this “liquid biopsy” difficult to capture and study. For this reason, there is continued interest in developing robust techniques for CTC isolation. Exploration into unique molecular signatures that can be used for effective enrichment is needed. To fortify the role of “liquid biopsies” and CTCs as a potential predictive biomarker in the clinical setting, focus should be placed on developing more precise and efficient CTC enrichment techniques.

## Current use of CTCs

### Trends in CTC enrichment techniques

In a liquid biopsy, there is roughly one CTC detectable out of ten million white blood cells per one milliliter of blood [2]. To add complexity, similar to a primary tumor, these circulating cells are known to exhibit heterogeneity with multiple subpopulations expressing different molecular markers [3]. The lack of a single ubiquitous marker or molecular signature present on all cancer cells creates a challenge in developing highly precise CTC isolation techniques. This has led to the invention of isolation devices that focus on exploiting epithelial marker proteins that are expressed on tumor cells and are deficient on the surrounding blood cells. In 2004, the U.S. Food and Drug Administration (FDA) approved an epithelial cell adhesion molecule (EpCAM)-dependent technique for use as a prognostic biomarker in breast cancer [4], which was then expanded for use in prostate and colorectal cancer patients [5]. EpCAM is an attractive target for CTC isolation because it is detectable on the majority of epithelial-derived cancer types and not detectable on leukocytes, making it possible to isolate CTCs effectively from other blood components [6]. Since its approval, the EpCAM-dependent device has become the “gold standard’ for CTC isolation.

**Fig. 1:**
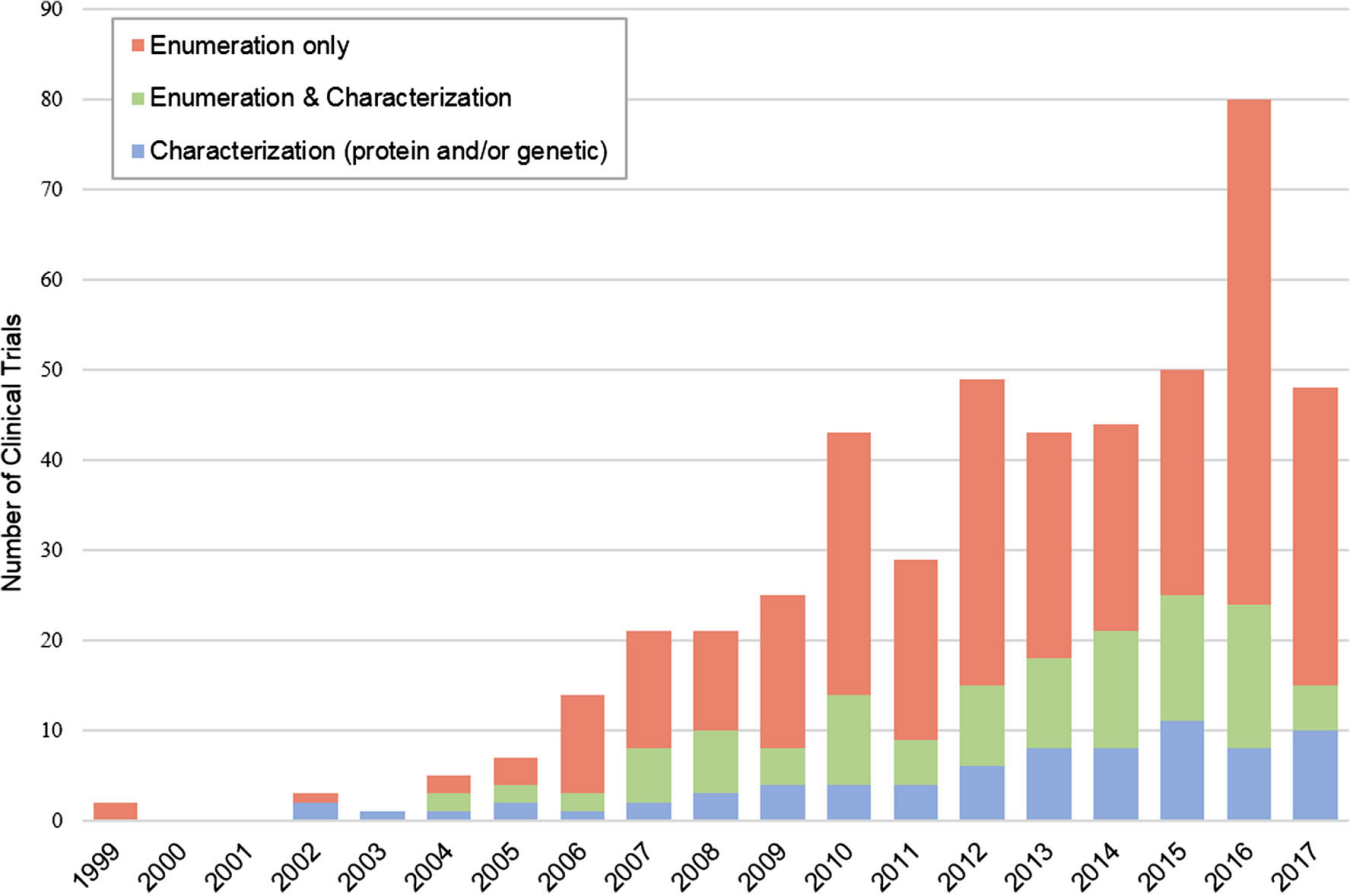
Trends in CTC evaluation. Number of clinical studies using each type of CTC evaluation between 1999 and 2017. Data were obtained from ClinicalTrials.gov by searching the terms “circulating tumor cells” and “cancer”, revealing 493 clinical programs involving CTC evaluation. The use of CTCs in clinical trials has been increasing over the past 20 years. These trials include enumeration, characterization, or a combination. Molecular characterization (blue), which encompasses genetic and proteomic characterization of CTCs, shows a steady increase and signals a trend in cancer therapeutic development

**Fig. 2:**
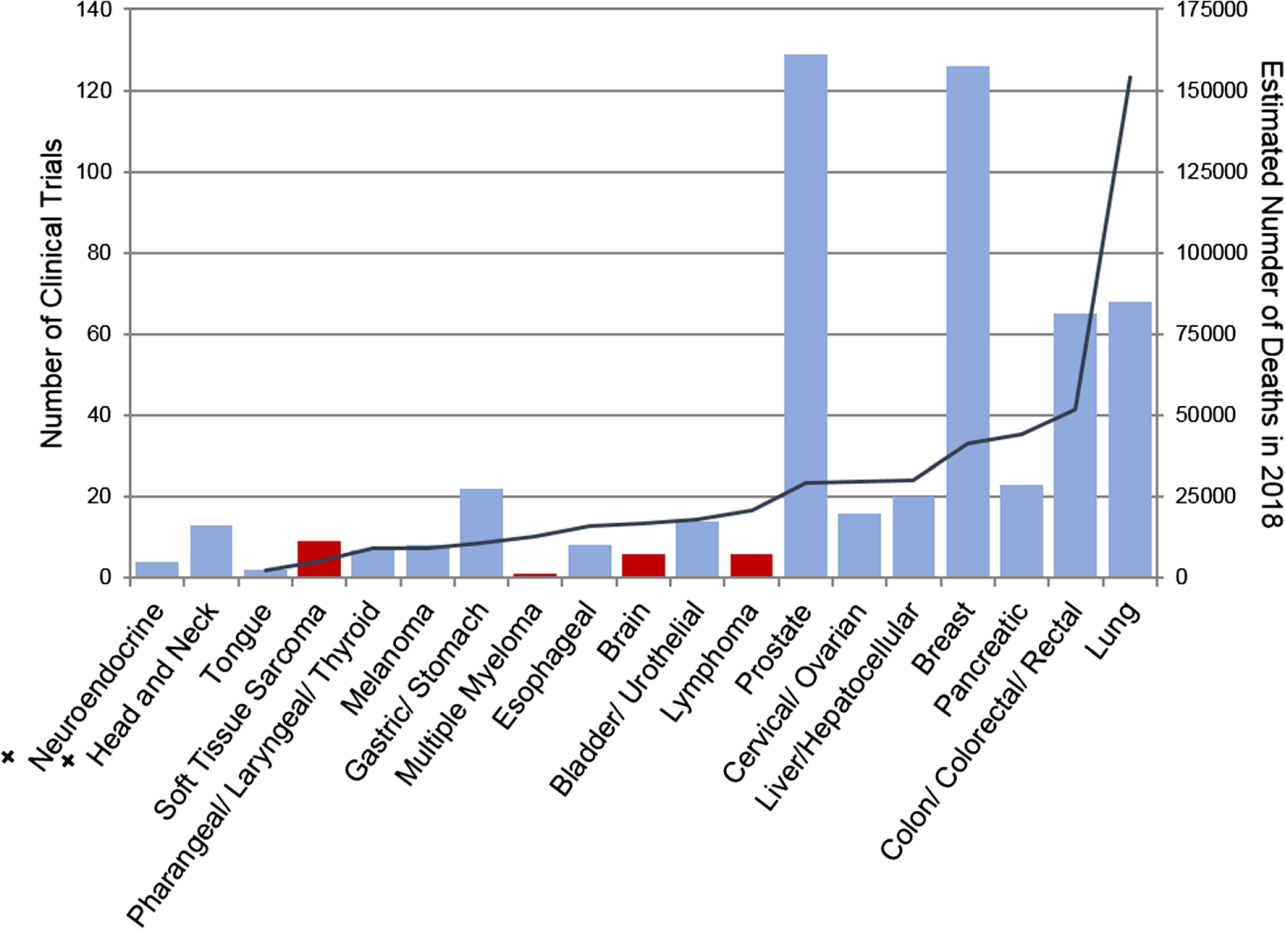
Exploration of CTCs in common cancers. Number of clinical trials conducted between 1999 and 2017 evaluating CTCs in common primary cancer types (left axis, bar graph). Primary cancer types are labeled in ascending order based on the estimated number of deaths in 2018 in the U.S. [7](right axis, line graph). Red bars indicate tumors of non-epithelial origin. +Incidence not reported, excluded from SEER common cancer list

Approval of a CTC enrichment device facilitated investigations into the clinical use of CTCs for monitoring disease progression and therapeutic response, resulting in a steady increase in the number of clinical studies measuring CTCs (Fig. 1). Despite this progress, clinical use of the EpCAM-dependent device remains rather limited. Over half of all clinical studies on CTCs since 1999 were conducted in only three cancer types: prostate, breast, and lung cancer (Fig. 2). Although the role of CTCs in other cancer types has been studied, their clinical use is yet to be confirmed due to various limitations in enrichment [8–11].

### Markers for CTC enrichment

Enrichment devices reliant on a specific cell surface marker for isolation can pose a bias by excluding sub-populations of CTCs that are deficient or express low levels of the marker. A key example is seen with EpCAM-dependent enrichment of CTCs; only CTCs that retain epithelial characteristics are isolated, excluding CTCs with mesenchymal traits. Cancer cells can biochemically alter their phenotype, undergoing epithelial to mesenchymal transition (EMT), gaining invasive mesenchymal and stem cell-like properties and losing epithelial characteristics, such as cell surface EpCAM expression. The CTC population can therefore be comprised of cells with a range of epithelial, epithelial-mesenchymal, and mesenchymal characteristics [12]. With EpCAM-dependent enrichment techniques, the marker-negative CTCs are undetectable, creating uncertainty in the accuracy of a patient’s CTC status. Analysis is then limited to cancers with CTCs that predominantly maintain epithelial characteristics.

Recent studies have focused on evaluating the epithelial and mesenchymal characteristics of CTCs to determine how these phenotypic differences can affect their clinical utility [13–16]. One such study assessing the clinical relevance of epithelial, mesenchymal and epithelial-mesenchymal CTCs in colorectal cancer found that only mesenchymal and epithelial-mesenchymal CTCs, not epithelial CTCs, correlated with clinical stage and metastasis [13]. Similarly, mesenchymal CTCs in esophageal squamous cell carcinoma have been shown to denote clinical stage and treatment efficacy [14]. These findings support the importance of isolating mesenchymal CTCs for inclusion in downstream analysis.

**Fig. 3:**
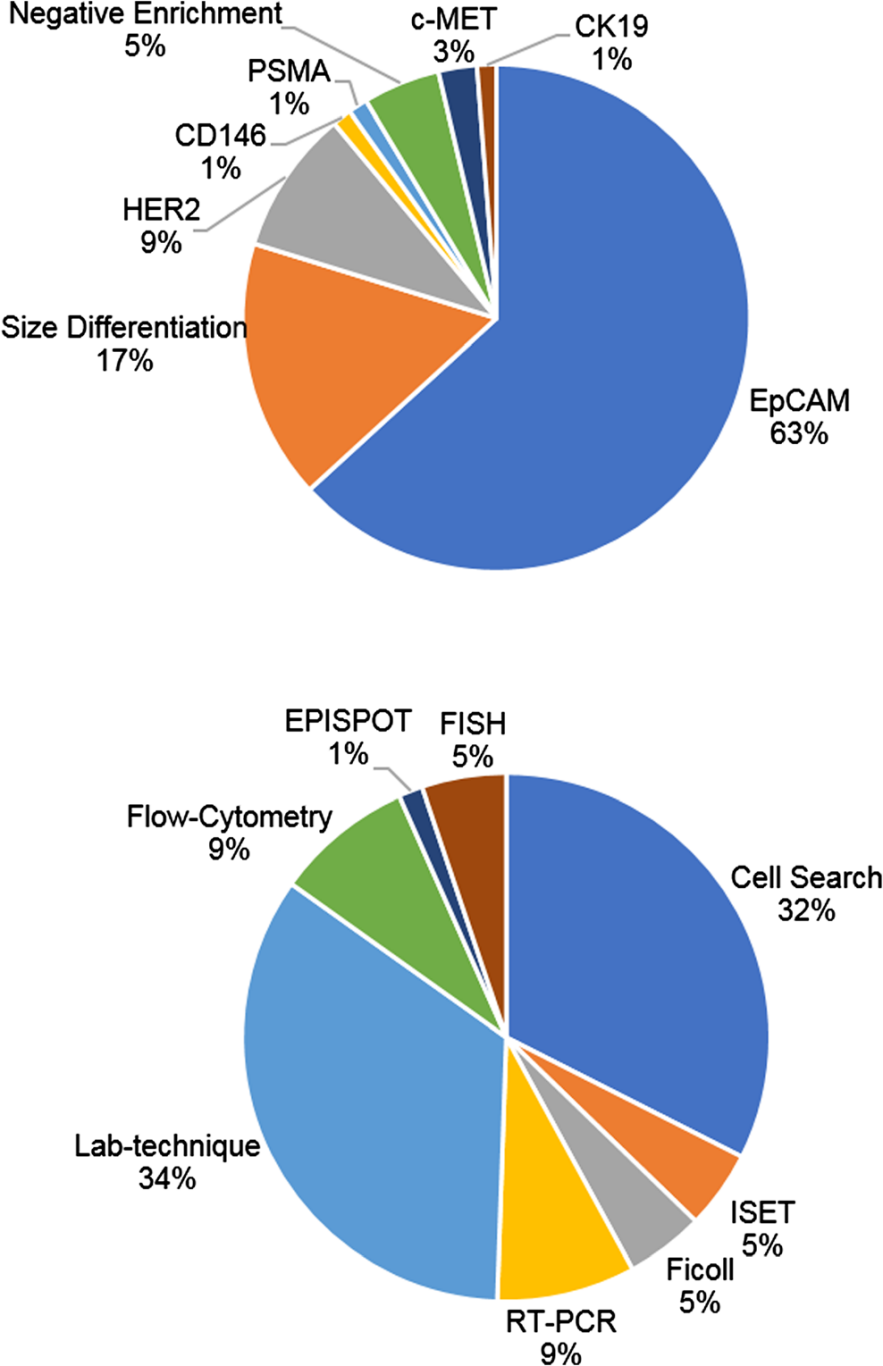
Proportion of clinical studies between 1999 and 2017 using specific isolation markers (top panel) and specific techniques for enrichment/analysis (bottom panel). Lab-technique was a classification used for clinical studies in which the disclosed device was unique/not commercially available. Abbreviations: epithelial cell adhesion molecule (EpCAM), human epidermal growth factor receptor 2 (HER2), cluster of differentiation 146 (CD146), prostate-specific membrane antigen (PSMA), cytokeratin 19 (CK19), isolation by size of tumor cells (ISET), reverse transcription polymerase chain reaction (RT-PCR), epithelial immunoSPOT assay (EPISPOT), fluorescence in situ hybridization (FISH)

Despite the challenge in addressing the totality of CTCs, EpCAM-dependent enrichment is still the most commonly used marker in CTC isolation (Fig. 3). More than eight EpCAM-dependent enrichment devices have been developed since 2004 for use in clinical trials (summarized in Table 1); with their use as prognostic tools pending confirmation.

**Table 1:** Common CTC enrichment techniques used in the clinical setting categorized by methodology. Clinical studies referenced exemplify typical use but do not encompass all the trials using this device between 1999 and 2017

	Device	Feature for isolation	Technology	Comments	Clinical studies
Positive enrichment	CellSearch (Veridex)	EpCAM	FDA approved, ferromagnetic beads labeled with EpCAM-antibodies, stained for CK^+^, CD45^−^, DAPI^+^	No detection of EMT, single marker, expensive, no further downstream analysis	[17, 18]
	GILUPI CellCollector	EpCAM	*In vivo* metal probe with anti-EpCAM antibodies on surface, high sensitivity	Invasive 30-min insertion, no detection of EMT, single marker, no further downstream analysis	NCT03071900, NCT02507778
	IsoFlux (Fluxion Biosciences)	EpCAM and optional additional markers	Antibody based magnetic separation by flow cytometry, additional markers available	Based on cell surface antigen presence, automated identification, incorporates Next Generation Sequencing	[19]
	Herringbone CTC-Chip	EpCAM	Antibody micropost capture, microfluidic device	Increased interactions with antibody surface, based on cell surface antigen presence, single marker, no detection of EMT	[20]
	MAC MicroBead Technology (Miltenyi)	EpCAM	Superparamagnetic nano-sized beads	No differentiation of low and high expression levels [21], single marker, no detection of EMT	[22]
	BD FACScanto II	EpCAM and CKs	Flow-cytometry antibody fluorescent separation	No detection of EMT, based on cell surface antigen presence	[23, 24]
	GEDI	EpCAM	Microfluidic device with EpCAM-antibody labeled obstacles, stained for CD45^−^, DAPI^+^, Pdx-1^+^	No detection of EMT, single marker, no further downstream analysis	[25]
	EPISPOT	Many cell surface proteins	CD45 depletion followed by immunofluorescence using fluorochrome labeled secondary antibodies	Utilize CTC secreted proteins for enrichment, protein characterization possible	[26]
Negative enrichment	RosetteSep CTC Enrichment Cocktail/ CD45 Depletion Cocktail (STEMCELL)	Density	RBC lysis with or without CD45 depletion followed by density gradient to remove lymphocytes and other unwanted cells	Requires downstream analysis to confirm CTC status	NCT02349867
	ApoStream	Dielectric property	Dielectrophoresis field-flow that repels blood cells into eluent	Takes advantage of CTC phenotypic and dielectric properties	NCT02349867
	MAC MicroBead Technology (Miltenyi)	CD45	Superparamagnetic nano-sized beads deplete CD45^+^ leukocytes	No differentiation of low and high expression levels [21]	
Size-based	CellSieve (Creatv Microtech)	Size	Biocompatible polymer filter with 7-μm diameter pores	Loss of small diameter CTCs, high leukocyte retention	
	ISET	Size	Polycarbon membrane with 8-μm diameter pores	Loss of small diameter CTCs, high leukocyte retention	[27, 28] NCT01776385
	Viator Therapeutic Oncophoresis System	Size and deformability	*Ex vivo* extracorporeal fluid circuit, cross-flow filtration, returns other blood elements back to the body	Time-consuming, patient compliance with procedure, loss of small diameter CTCs	NCT01943500
	Parosotix System (ANGEL)	Size and deformability	Microfluidic device, separates cells when flowing through steps of the cassette		NCT02490800, NCT03427450, NCT02785731
	CanPatrol (SurExam)	Size	RBC lysis followed by filtration in an 8-μm diameter porous membrane	Loss of small diameter CTCs, high leukocyte retention	[14, 29] NCT02951897
	ScreenCell Cyto Filtration	Size	8-μm diameter porous membrane		[30]
Density-based	Ficoll-Paque	Density	Reagent separates peripheral blood mononuclear cells for downstream analysis of CTCs	Requires downstream analysis to confirm CTC status	[31]

*CK, cytokeratin; DAPI, 4′,6-diamidino-2-phenylindole

Alternative approaches are being developed to attempt collection of a phenotypically representative sample of the entire CTC population (Fig. 3), such as label-free devices that use size differentiation to capture CTCs or methods, such as negative depletion, which use immunomagnetic and microfluidic removal of blood cells leaving only the CTCs for downstream processing (Table 1). Size-based devices take advantage of large tumor cell diameters (> 8 μm) by using a porous filter with a size range to allow small diameter leukocytes and other blood elements to pass through [32]. Such a filtration approach is effective in isolating the diverse population of CTCs; yet, the inefficient depletion of white blood cells often leaves substantial background in the isolate, making downstream analysis difficult. Negative depletion typically involves red blood cell (RBC) lysis and removal of other blood elements by anti-CD45 immunomagnetic separation of leukocytes, allowing label-free CTC isolation. Following enrichment, immunocytochemistry is a common procedure to distinguish CTCs from cells in the peripheral blood stream not properly excluded during the isolation. This imaging technique often involves fixation and permeabilization, potentially interfering with further downstream molecular analysis of CTCs.

### Clinical utility of CTCs

CTC enumeration is currently used as a prognostic marker of progression-free survival (PFS) and overall survival (OS) in breast, colorectal, and prostate cancer [33]. When using CTCs as a surrogate biomarker, a minimum cutoff value is designated to evaluate prognostic outcome. However, this cutoff value varies across isolation devices, the protein marker used, and the origin of the primary tumor. For example, ≥ 5 CTCs in 7.5 mL of blood was determined retrospectively to be a cutoff value to indicate poor prognosis based on OS and PFS in metastatic breast cancer patients when using an EpCAM-dependent device [34]. These findings established this cutoff value as “CTC-positivity,” which is now widely used for prognostication in breast cancer [35]. Similar retrospective determination of CTC-cutoff values were found in clinical trials for metastatic prostate cancer, which found use of ≥ 5 CTCs as a cutoff based on correlation with OS [36] and colorectal cancer (CRC), in which > 3 CTCs was associated with OS and PFS [37]. While correlations between enumeration and survival rates have been determined [38], the optimal cutoff values vary across trials and is limited to epithelial-marker positive cancers [12, 32, 39–41]. Studies on other cancer types have difficulty defining a prognostic cutoff value [9] showing necessity for more effective CTC isolation and retrospective analyses.

In addition to use as a prognostic biomarker, CTC enumeration is being used as an exploratory indicator of efficacy and a measurement of treatment response [42–44]. A drop-off in CTC counts showed promise as an early efficacy endpoint in a study observing cabazitaxel response in metastatic castration resistant prostate cancer (mCRPC) patients with docetaxel resistance [42]. Enumeration was also tested as a patient stratification biomarker in the SWOG S0500 trial, which used changes in CTC counts in response to therapy as an early indicator of therapy resistance to determine a change in treatment regimen, though this prospective use was ineffective at prolonging OS in metastatic breast cancer patients [45]. To strengthen the use of CTCs as a biomarker, enumeration is being used as a secondary outcome measure to increase the accuracy of other prognostic markers used in clinical practice, such as tumor associated protein markers (i.e., PSA, CEA, CA125) and imaging tests. One study specifically found that using CTC enumeration in conjunction with positron-emission computerized tomography (PET-CT) had greater prognostic significance than either measure on its own [31].

To explore the predictive value of CTCs in the clinical setting, there must be uniformity in isolation and characterization methods. Without uniformity, there is inconsistency in the CTC population analyzed [46]—whether it be phenotypic variability or inconsistent capture efficiency in cancer types—limiting the scope of any significant findings. Clinical validation of markers identified in ongoing retrospective studies [47] will help to clarify how CTCs can be used to direct personalized medicine and drug development.

### Molecular characterization

Interest in molecular characterization, specifically genetic and proteomic characterization, has been on the rise as the clinical utility of CTCs for therapy personalization has become more apparent (Fig. 1, Fig. 3). Genetic characterization explores the presence of a specific mutation or activation of an oncogene that can be targeted through therapy or correlated with progression or response. NCT03366116 is a clinical trial in progress exploring the efficacy of the nucleoside analog abbreviated Aza-TdC intended to upregulate tumor suppressor genes, which will be monitored in isolated CTCs [48]. Whole transcriptome sequencing of CTCs can identify specific genetic events that occur during the metastatic process and therapeutic response, holding promise for cancer drug development [49]. Technical challenges within CTC capture efficiency have made progress in this field difficult, limiting the number of genomic studies and significant findings to date [50]. Sequencing methods, like mRNA-Seq, require high capture purity and viable CTCs for downstream analysis, which is difficult to achieve with current isolation devices due to the intensive fixation and labeling methods used [51]. One study was able to identify a CTC gene signature that could predict survival in pancreatic cancer patients using a unique method of negative depletion followed by gene microarray analysis [52].

Detecting the presence of specific proteins on the surface of CTCs is more commonly used and can be done by incorporating additional protein labeling into the isolation-devices’ standard immunocytochemistry (including CD45, DAPI, cytokeratins, and EpCAM). The ongoing DETECT studies (III, IV, and V) incorporate human epidermal growth factor receptor 2 (HER2) staining into an EpCAM-dependent protocol to identify HER2-positive CTCs in patients with HER2-negative primary tumors [53]. Patients with HER2-negative breast cancer that are found to have HER2-positive CTCs are allocated to the DETECT III study where efficacy of HER2-targeted therapy is observed as a means of controlling the metastatic process [54]. An alternative study using the Pro Onc Assay (Prometheus Laboratories) to identify HER2-positive CTCs in HER2-negative metastatic breast cancer patients found that HER2-targeted therapy was not effective in this population due to continued disease progression [55]. This discordance between primary tumor and CTC phenotypes may contribute to recurrence and disease progression. For this reason, biopsy and analysis of the primary tumor must still be used in conjunction with CTC molecular characterization for accurate determination of guided therapies. The intrinsic heterogeneity of protein expression across the CTC population and between the primary and circulating tumor populations makes the use of CTCs as a “liquid biopsy” for real-time monitoring of cancer lesions and targeted treatment response challenging [56].

With CTC heterogeneity posing a challenge to understanding and targeting the metastatic process, identification of a more commonly expressed molecular marker and development of techniques that utilize such a marker is imperative. For biomarker discovery, label-free devices and techniques that leave CTCs relatively “untouched” for downstream analysis, such as size filtration techniques, can be used. Recently, a novel cancer cell-specific surface marker was identified and was shown to increase the efficiency and specificity of CTC isolation from patient blood samples. This marker, a unique proteoglycan modification of chondroitin sulfate (CS) called oncofetal CS (ofCS), was found to be expressed on 95% of patient-derived human cancer cell lines of hematopoietic, epithelial, and mesenchymal origin [57]. CELLection beads and microfluidics designed to bind ofCS were stated to be capable of isolating CTCs with high efficiency [39]. ofCS is present on cancer cell types independent of origin or disease stage, meaning it exists on EpCAM-negative and EMT CTCs as well [39]. Identification of a CTC-specific surface marker, such as ofCS, may eliminate the need for subtype specific markers in CTC isolation, reducing current concerns with establishing consistency in CTC clinical use and significance.

## Perspectives

Understanding the role CTCs play in the metastatic process can aid the development of anticancer therapies and improve disease evaluation [58]. The current state of CTC isolation strategies underlines the need for more reliable enrichment techniques that can isolate the entire CTC population and be combined with downstream molecular characterization. These techniques may include more ubiquitously expressed markers or molecular signatures on CTCs, like ofCS, to investigate their clinical utility and application in different cancer types. The identification of a more global signature for CTC identification and enumeration can eliminate many of the inconsistencies across methods, improving the isolation and targeting of this metastatic population. Implementation of clinical guidelines for CTC studies has been proposed as a method of improving standardization within trials with respect to experimental design and analysis [59]. Once a guideline for CTC clinical studies has become well established, clinical implementation of CTCs as markers of progression or treatment response can be more clearly defined.

Heterogeneity in CTC phenotype and discordance with primary tumor type also needs to be addressed. The process of EMT highlights how CTC phenotype evolves and can provide valuable insight into the metastatic process, disease progression, and chemotherapy resistance. Molecular characterization holds promise for personalized cancer therapy, not only providing a potential target for therapeutic intervention, but also a reliable and consistent means of isolating the entire CTC population. CTC characterization used prospectively in treatment decision and drug development could reshape cancer therapeutic practices.

## References

[1] Seyfried TN, Huysentruyt LC (2013). On the origin of cancer metastasis. Critical Reviews in Oncogenesis.

[2] Moon DH, Lindsay DP, Hong S, Wang AZ (2018). Clinical indications for, and the future of, circulating tumor cells. Advanced Drug Delivery Reviews.

[3] Marusyk A, Polyak K (2010). Tumor heterogeneity: causes and consequences. Biochimica et Biophysica Acta.

[4] Administration, F. a. D. (2005). 510(k) substantial equivalence determination decision summary- Veridex, LLC (K050245).

[5] Administration, F. a. D. (2010). Special 510(k) Device modification ODE review memorandum- Veridex, LLC (K103502).

[6] Wit S d, Dalum G v, Lenferink ATM, Tibbe AGJ, Hiltermann TJN, Groen HJM, van Rijn CJM, Terstappen LWMM (2015). The detection of EpCAM+ and EpCAM– circulating tumor cells. Scientific Reports.

[7] Noone, A., Howlader, N., Krapcho, M., Miller, D., Brest, A., Yu, M., et al. *SEER Cancer Statistics Review, 1975–2015*. Bethesda, MD: National Cancer Institute.

[8] Donepudi S, Reisinger SA, McGregor JR, Tharkar S, Samlowski S, Ostler D, Shen S, Samlowski WE (2013). Circulating tumor cell cultures as a predictive marker during salvage therapy of refractory Merkel cell carcinoma with chemotherapy and electron beam radiation. Journal of Cancer Therapy.

[9] Flaig TW, Wilson S, van Bokhoven A, Varella-Garcia M, Wolfe P, Maroni P, Genova EE, Morales D, Lucia MS (2011). The detection of circulating tumor cells in metastatic and clinically localized urothelial carcinoma. Urology.

[10] Li Y, Gong J, Zhang Q, Lu Z, Gao J, Li Y, Cao Y, Shen L (2016). Dynamic monitoring of circulating tumour cells to evaluate therapeutic efficacy in advanced gastric cancer. [clinical study]. British Journal of Cancer.

[11] Reeh M, Effenberger KE, Koenig AM, Riethdorf S, Eichstädt D, Vettorazzi E, Uzunoglu FG, Vashist YK, Izbicki JR, Pantel K, Bockhorn M (2015). Circulating tumor cells as a biomarker for preoperative prognostic staging in patients with esophageal Cancer. Annals of Surgery.

[12] Barrière G, Tartary M, Rigaud M (2012). Epithelial mesenchymal transition: a new insight into the detection of circulating tumor cells. ISRN Oncology.

[13] Zhao R, Cai Z, Li S, Cheng Y, Gao H, Liu F, Wu S, Liu S, Dong Y, Zheng L, Zhang W, Wu X, Yao X (2017). Expression and clinical relevance of epithelial and mesenchymal markers in circulating tumor cells from colorectal cancer. Oncotarget.

[14] Chen W, Li Y, Yuan D, Peng Y, Qin J (2018). Practical value of identifying circulating tumor cells to evaluate esophageal squamous cell carcinoma staging and treatment efficacy. Thorac Cancer.

[15] NCT01866202: study of circulating and tumor biomarkers in breast cancer patients (2013). ClinicalTrials.gov.

[16] NCT03156777: Application value of CTCs detection for advanced gastric cancer patients (2017). ClinicalTrials.gov.

[17] de Bono JS, Scher HI, Montgomery RB, Parker C, Miller MC, Tissing H, Doyle GV, Terstappen LWWM, Pienta KJ, Raghavan D (2008). Circulating tumor cells predict survival benefit from treatment in metastatic castration-resistant prostate cancer. Clinical Cancer Research.

[18] Larsson A-M, Jansson S, Bendahl P-O, Levin Tykjaer Jörgensen C, Loman N, Graffman C, Lundgren L, Aaltonen K, Rydén L (2018). Longitudinal enumeration and cluster evaluation of circulating tumor cells improve prognostication for patients with newly diagnosed metastatic breast cancer in a prospective observational trial. Breast Cancer Research.

[19] Gonzalez-Rivera M, Picornell AC, Alvarez EL, Martin M (2016). A cross-sectional comparison of druggable mutations in primary tumors, metastatic tissue, circulating tumor cells, and cell-free circulating DNA in patients with metastatic breast Cancer: The MIRROR Study Protocol. JMIR Research Protocols.

[20] Sundaresan TK, Sequist LV, Heymach JV, Riely GJ, Jänne PA, Koch WH (2016). Detection of T790M, the acquired resistance EGFR mutation, by tumor biopsy versus noninvasive blood-based analyses. Clinical Cancer Research.

[21] Millner LM, Linder MW, Valdes R (2013). Circulating tumor cells: a review of present methods and the need to identify heterogeneous phenotypes. Annals of Clinical and Laboratory Science.

[22] Shi J, Li Y, Liang S, Zeng J, Liu G, Mu F, Li H, Chen J, Liu T, Niu L (2016). Analysis of circulating tumor cells in colorectal cancer liver metastasis patients before and after cryosurgery. Cancer Biology & Therapy.

[23] Milojkovic Kerklaan B, Pluim D, Bol M, Hofland I, Westerga J, van Tinteren H, Beijnen JH, Boogerd W, Schellens JHM, Brandsma D (2016). EpCAM-based flow cytometry in cerebrospinal fluid greatly improves diagnostic accuracy of leptomeningeal metastases from epithelial tumors. Neuro-Oncology.

[24] Qin Z, Chen J, Zeng J, Niu L, Xie S, Wang X, Liang Y, Wu Z, Zhang M (2017). Effect of NK cell immunotherapy on immune function in patients with hepatic carcinoma: a preliminary clinical study. Cancer Biology & Therapy.

[25] Brown JC, Troxel AB, Ky B, Damjanov N, Zemel BS, Rickels MR, Rhim AD, Rustgi AK, Courneya KS, Schmitz KH (2016). A randomized phase II dose-response exercise trial among colon cancer survivors: purpose, study design, methods, and recruitment results. Contemporary Clinical Trials.

[26] Cayrefourcq L, Mazard T, Joosse S, Solassol J, Ramos J, Assenat E, Schumacher U, Costes V, Maudelonde T, Pantel K, Alix-Panabieres C (2015). Establishment and characterization of a cell line from human circulating colon cancer cells. Cancer Research.

[27] Abdallah EA, Fanelli MF, Souza e Silva V, Machado Netto MC, Gasparini Junior JL, Araújo DV, Ocea LM, Buim ME, Tariki MS, Alves VD, Piana de Andrade V, Dettino AL, Abdon Lopes de Mello C, Chinen LT (2016). MRP1 expression in CTCs confers resistance to irinotecan-based chemotherapy in metastatic colorectal cancer. International Journal of Cancer.

[28] Gemenetzis G, Groot VP, Yu J, Ding D, Teinor JA, Javed AA, Wood LD, Burkhart RA, Cameron JL, Makary MA, Weiss MJ, He J, Wolfgang CL (2018). Circulating tumor cells dynamics in pancreatic adenocarcinoma correlate with disease status: results of the prospective CLUSTER study. Annals of Surgery.

[29] Yang L, Lv Z, Xia W, Zhang W, Xin Y, Yuan H, Chen Y, Hu X, Lv Y, Xu Q, Weng X, Ni C (2018). The effect of aspirin on circulating tumor cells in metastatic colorectal and breast cancer patients: a phase II trial study. Clinical and Translational Oncology.

[30] Sefrioui D, Blanchard F, Toure E, Basile P, Beaussire L, Dolfus C, Perdrix A, Paresy M, Antonietti M, Iwanicki-Caron I, Alhameedi R, Lecleire S, Gangloff A, Schwarz L, Clatot F, Tuech JJ, Frébourg T, Jardin F, Sabourin JC, Sarafan-Vasseur N, Michel P, di Fiore F (2017). Diagnostic value of CA19.9, circulating tumour DNA and circulating tumour cells in patients with solid pancreatic tumours. [molecular diagnostics]. British Journal of Cancer.

[31] Ma B, King AD, Leung L, Wang K, Poon A, Ho WM, Mo F, Chan CML, Chan ATC, Wong SCC (2017). Identifying an early indicator of drug efficacy in patients with metastatic colorectal cancer—a prospective evaluation of circulating tumor cells, 18F-fluorodeoxyglucose positron-emission tomography and the RECIST criteria. Annals of Oncology.

[32] Hosokawa M, Kenmotsu H, Koh Y, Yoshino T, Yoshikawa T, Naito T, Takahashi T, Murakami H, Nakamura Y, Tsuya A, Shukuya T, Ono A, Akamatsu H, Watanabe R, Ono S, Mori K, Kanbara H, Yamaguchi K, Tanaka T, Matsunaga T, Yamamoto N (2013). Size-based isolation of circulating tumor cells in lung Cancer patients using a microcavity Array system. PLoS One.

[33] (FDA), F. a. D. A (2004). 510(k) substantial equivalence determination decision summary - Veridex, LLC (K040898).

[34] Cristofanilli M, Budd GT, Ellis MJ, Stopeck A, Matera J, Miller MC, Reuben JM, Doyle GV, Allard WJ, Terstappen LWMM, Hayes DF (2004). Circulating tumor cells, disease progression, and survival in metastatic breast cancer. New England Journal of Medicine.

[35] Helissey C, Berger F, Cottu P, Diéras V, Mignot L, Servois V, Bouleuc C, Asselain B, Pelissier S, Vaucher I, Pierga JY, Bidard FC (2015). Circulating tumor cell thresholds and survival scores in advanced metastatic breast cancer: the observational step of the CirCe01 phase III trial. Cancer Letters.

[36] Moreno JG, Miller MC, Gross S, Allard WJ, Gomella LG, Terstappen LWMM (2005). Circulating tumor cells predict survival in patients with metastatic prostate cancer. Urology.

[37] Cohen SJ, Punt CJA, Iannotti N, Saidman BH, Sabbath KD, Gabrail NY, Picus J, Morse M, Mitchell E, Miller MC, Doyle GV, Tissing H, Terstappen LWMM, Meropol NJ (2008). Relationship of circulating tumor cells to tumor response, progression-free survival, and overall survival in patients with metastatic colorectal cancer. Journal of Clinical Oncology.

[38] (2010). Circulating tumour cells. *Nature Reviews Cancer, 11*, 3. 10.1038/nrc3000.

[39] Agerbaek MO, Bang-Christensen SR, Yang MH, Clausen TM, Pereira MA, Sharma S (2018). The VAR2CSA malaria protein efficiently retrieves circulating tumor cells in an EpCAM-independent manner. Nature Communications.

[40] Goodman OB, Fink LM, Symanowski JT, Wong B, Grobaski B, Pomerantz D (2009). Circulating tumor cells in patients with castration-resistant prostate cancer baseline values and correlation with prognostic factors. Cancer Epidemiology Biomarkers; Prevention.

[41] Heller G, McCormack R, Kheoh T, Molina A, Smith MR, Dreicer R, Saad F, de Wit R, Aftab DT, Hirmand M, Limon A, Fizazi K, Fleisher M, de Bono JS, Scher HI (2018). Circulating tumor cell number as a response measure of prolonged survival for metastatic castration-resistant prostate cancer: a comparison with prostate-specific antigen across five randomized phase III clinical trials. Journal of Clinical Oncology.

[42] Anido U, Fita MJJ, Munielo-Romay L, Pérez-Valderrama B, Mellado B, de Olza MO, Calvo OF, Castellano D, Parra EMF, Domenec M, Hernando S, Arija JA, Caballero C, Duran I, Campayo M, Climent MA (2016). Phase II study of weekly cabazitaxel for ‘unfit’ metastatic castration resistant prostate cancer patients (mCRPC) progressing after docetaxel (D) treatment. Circulating tumour cell (CTC) analysis (SOGUG-CABASEM Trial). Annals of Oncology.

[43] NCT02538432: phase II trial of EP4 receptor antagonist, AAT-007 (RQ-07; CJ-023,423) in advanced solid tumors (2015). ClinicalTrials.gov.

[44] Eigl BJ, Chi K, Tu D, Hotte SJ, Winquist E, Booth CM, Canil C, Potvin K, Gregg R, North S, Zulfiqar M, Ellard S, Ruether JD, le L, Kakumanu SA, Salim M, Allan AL, Feilotter H, Theis A, Seymour L (2018). A randomized phase II study of pelareorep and docetaxel or docetaxel alone in men with metastatic castration resistant prostate cancer: CCTG study IND 209. Oncotarget.

[45] Smerage JB, Barlow WE, Hortobagyi GN, Winer EP, Leyland-Jones B, Srkalovic G, Tejwani S, Schott AF, O'Rourke MA, Lew DL, Doyle GV, Gralow JR, Livingston RB, Hayes DF (2014). Circulating tumor cells and response to chemotherapy in metastatic breast cancer: SWOG S0500. Journal of Clinical Oncology.

[46] Lianidou ES, Markou A, Strati A (2012). Molecular characterization of circulating tumor cells in breast cancer: challenges and promises for individualized cancer treatment. Cancer and Metastasis Reviews.

[47] Wang D, Liu X, Hsieh B, Bruce R, Somlo G, Huang J, Sambucetti L (2015). Exploring glycan markers for immunotyping and precision-targeting of breast circulating tumor cells. Archives of Medical Research.

[48] NCT03366116: 5-aza-4′-Thio-2′-deoxycytidine (Aza-TdC) in people with advanced solid tumors (2017). ClinicalTrials.gov.

[49] Pantel K, Alix-Panabières C (2017). Circulating tumour cells and cell-free DNA in gastrointestinal cancer. Nature Reviews Gastroenterology &Amp; Hepatology.

[50] Magbanua MJM, Park JW (2014). Advances in genomic characterization of circulating tumor cells. Cancer and Metastasis Reviews.

[51] Chen, X. X., & Bai, F. (2015). Single-cell analyses of circulating tumor cells. In *Cancer biol med* (Vol. 12, pp. 184–192, Vol. 3). 10.7497/j.issn.2095-3941.2015.0056 PMC4607822 26487963

[52] Sergeant G, van Eijsden R, Roskams T, Van Duppen V, Topal B (2012). Pancreatic cancer circulating tumour cells express a cell motility gene signature that predicts survival after surgery. BMC Cancer.

[53] Fehm T, Müller V, Aktas B, Janni W, Schneeweiss A, Stickeler E, Lattrich C, Löhberg CR, Solomayer E, Rack B, Riethdorf S, Klein C, Schindlbeck C, Brocker K, Kasimir-Bauer S, Wallwiener D, Pantel K (2010). HER2 status of circulating tumor cells in patients with metastatic breast cancer: a prospective, multicenter trial. Breast Cancer Research and Treatment.

[54] Polasik A, Schramm A, Friedl TWP, Rack BK, Trapp EK, Fasching PA, Taran FA, Hartkopf AD, Schneeweiss A, Mueller V, Aktas B, Pantel K, Meier-Stiegen F, Wimberger P, Janni W, Fehm TN (2016). The DETECT study concept: individualized therapy of metastatic breast cancer. Journal of Clinical Oncology.

[55] Hainsworth JD, Murphy PB, Alemar JR, Daniel BR, Young RR, Yardley DA (2016). Use of a multiplexed immunoassay (PRO Onc assay) to detect HER2 abnormalities in circulating tumor cells of women with HER2-negative metastatic breast cancer: Lack of response to HER2-targeted therapy. Breast Cancer Research and Treatment.

[56] Bidard FC, Cottu P, Dubot C, Venat-Bouvet L, Lortholary A, Bourgeois H (2017). 117PAnti-HER2 therapy efficacy in HER2-negative metastatic breast cancer with HER2-amplified circulating tumor cells: results of the CirCe T-DM1 trial. Annals of Oncology.

[57] Salanti A, Clausen TM, Agerbaek MO, Al Nakouzi N, Dahlback M, Oo HZ (2015). Targeting human cancer by a glycosaminoglycan binding malaria protein. Cancer Cell.

[58] Caixeiro NJ, Kienzle N, Lim SH, Spring KJ, Tognela A, Scott KF, de Souza P, Becker TM (2014). Circulating tumour cells—a bona fide cause of metastatic cancer. Cancer and Metastasis Reviews.

[59] Bünger S, Zimmermann M, Habermann JK (2015). Diversity of assessing circulating tumor cells (CTCs) emphasizes need for standardization: a CTC guide to design and report trials. Cancer and Metastasis Reviews.

